# Tobamoviruses can be frequently present in the oropharynx and gut of infants during their first year of life

**DOI:** 10.1038/s41598-020-70684-w

**Published:** 2020-08-12

**Authors:** Yarenci Aguado-García, Blanca Taboada, Patricia Morán, Xaira Rivera-Gutiérrez, Angélica Serrano-Vázquez, Pavel Iša, Liliana Rojas-Velázquez, Horacio Pérez-Juárez, Susana López, Javier Torres, Cecilia Ximénez, Carlos F. Arias

**Affiliations:** 1 Instituto de Biotecnología, Universidad Nacional Autónoma de México, Av. Universidad 2001, 62210 Cuernavaca, Morelos Mexico; 2 Unidad de Investigación en Medicina Experimental, Facultad de Medicina, Universidad Nacional Autónoma de México, Dr. Balmis Num. 148 Doctores, 06726 Ciudad de México, Mexico; 3 Unidad de Investigación Médica en Enfermedades Infecciosas y Parasitarias, Hospital Pediatría, Centro Médico Nacional Siglo XXI, Instituto Mexicano del Seguro Social, 06726 Cuauhtémoc, Ciudad de México, Mexico

**Keywords:** Microbial communities, Virology

## Abstract

Plant viruses have been reported to be common in the gut of human adults, presumably as result of food ingestion. In this work, we report that plant viruses can also be found frequently in the gut and oropharynx of children during their first year of life, even when they are exclusively breast-fed. Fecal and oropharynx samples were collected monthly, from birth to 1 year of age, from three apparently healthy children in a semi-rural community and analyzed by next generation sequencing. In 100% of the fecal samples and 65% of the oropharynx samples at least one plant virus was identified. Tobamoviruses in the *Virgaviridae* family were by far the most frequently detected, with tropical soda apple mosaic virus, pepper mild mottle virus, and opuntia tobamovirus 2 being the most common species. Seventeen complete virus genomes could be assembled, and phylogenetic analyses showed a large diversity of virus strains circulating in the population. These results suggest that children are continuously exposed to an extensive and highly diverse collection of tobamoviruses. Whether the common presence of plant viruses at an early age influences the infant’s immune system, either directly or through interaction with other members of the microbiota, remains to be investigated.

## Introduction

Plant viruses are known to be abundant and ubiquitous in nature. Despite this, their study has been mostly focused to pathogenic viruses of crop plants, while the great majority of the phytobiome viruses remain understudied^1,2^. Animals, including humans, are frequently exposed to these viruses and it is not surprising that characterization of the human gut virome has shown that in addition to bacterial and mammalian viruses, plant pathogenic viruses are also commonly found in feces of healthy adults^3,4^. These viruses can also be found in a number of processed foods and, therefore, are considered to be of dietary origin.

Metagenomic studies describing the composition of eukaryotic viruses in the gastrointestinal and respiratory tracts of children are limited, and only a few of them have included children under 1 year of age^5–11^. In addition, most of the reported studies have characterized the virome in diseased children^8–11^ and just a few have studied community-based healthy children^5–7^. Therefore, we aimed to determine the diversity and dynamic of the oropharyngeal and gastrointestinal eukaryotic viromes in children under 1 year of age in a semi-rural population in Mexico, using next generation sequencing. In addition to human viruses, we found that plant viruses were commonly present in the gut and the oropharynx of children during their first year of life and, surprisingly, they were found as early as 2-weeks after birth in exclusively breast-fed infants. Tobamoviruses, in the *Virgaviridae* family, were the most abundant, and were present in most of the samples analyzed. Of interest, antibodies to plant viruses have been found in animals, including humans^3^, and it has also been shown that cowpea mosaic virus can disseminate systemically when orally administered to mice^12^. Whether the common presence of these viruses at an early age has an effect in the infant’s immune system and maturation of the gut remains to be investigated.

## Results

### Study population and sample analysis

**Figure 1:**
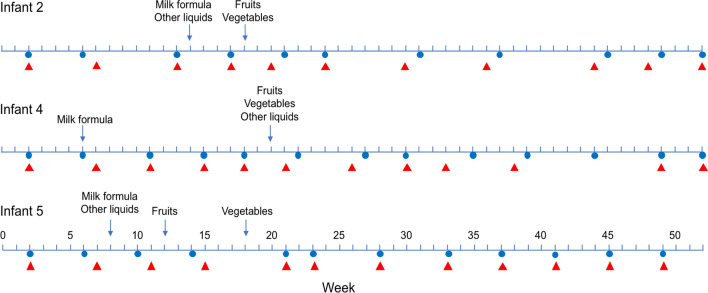
Overview of fecal and oropharynx sample collection from the three children enrolled in the study. Blue circles, feces collection; red triangle, oropharynx swab collection. The week when the children started to ingest foods different from breast-milk is indicated.

Fecal and oropharynx samples from three apparently healthy children, with no respiratory or gastrointestinal symptoms during the study, were collected monthly, starting 2 weeks after birth and until 12 months of age (Fig. 1). Single oropharyngeal and fecal samples were collected from their mothers about 1 month before child delivery. General information for the participating infants and their mothers is described in Tables 1 and 2.

**Table 1:** General information of children.

Variable	Infant 2	Infant 4	Infant 5
**Perinatal and clinical history**			
Gender	Male	Male	Female
Gestation weeks	41 weeks	42 weeks	38 weeks
Type of birth	Vaginal delivery	Cesarean section	Cesarean section
Frequency of perinatal follow-up	Monthly	Monthly	Monthly
Birth weight and height	3.570 kg/49 cm	3.300 kg/48 cm	2.630 kg/47 cm
Vaccination scheme	Complete scheme	Complete scheme	Complete scheme
Genetic background	Diabetes and obesity	None	None
Diseases	No	No	No
Use of antibiotics	No	No	No
**Children’s diet**			
Breastfeeding	Maternal breast and milk formula	Maternal breast and milk formula	Maternal breast and milk formula
Supplementary feeding	Water and infusions (after 4 months)	Infusions and water (before 4 months)	Infusions (after 3 months)
Complementary solid food	Purees (vegetables, legumes, fruits, red meats) water, atoles	Purees (shallots, legumes, apple), yogurth, atoles	Purees (vegetables, legumes, fruits), atoles
**Habits of coexistence**			
The child lives with:	Parents	Parents	Parents
The child sleeps with:	Father	Grandmother	Parents
Who takes care of the child?	Father	Grandmother	Mother
The child plays with:	Sisters	Grandmother	Sister
**Sociodemographic and socioeconomic characteristics**			
Housing type material	Walls: adobe Ceilings: Foil Floor: soil	Walls: cardboard Ceiling: cardboard Floor: cement	Walls: brick Ceilings: cement Floor: soil
Services	Piped water, electricity, septic tank	Piped water, electricity, septic tank	Piped water, drainage and electricity
Technology	Cell phone and television	Cell phone	Cell phone
Father’s occupation	Does not work	Field worker	Builder
Mother’s occupation	Domestic worker	Housewife	Housewife
Health service	Public	Public	Public
Ethnicity	Mestizo	Mestizo	Mestizo
Animal/pet exposures	Yes (dog, cat and rooster)	Yes (dog)	Yes (dog)
Socioeconomic level (SL)	SL-E^1^	SL-E	SL-E

^1^SL-E: In this classification level, the majority (95%) of household heads have an elementary school education; available internet at home is minimal (0.1%); most of the salary is allocated to food (52%); and less than 5% of income is dedicated to child’s education.

**Table 2:** Characteristics of the participating mothers.

Variable	Mother 2	Mother 4	Mother 5
Mother’s age at the birth of child (years)	24	18	21
Feeding	Green peppers, tomatoes, legumes, white meats, fruits, dairy, water, infusions, atoles, soda, fried foods	Green peppers, tomatoes, white meat, fruits, dairy, atoles, water, infusions	Green peppers, tomatoes, legumes, red and white meats, dairy, water, infusions, atoles, soft drinks, fried foods, few fruit
Smoking	No	No	No
Alcoholism	No	No	No
Diseases	No	No	No

**Figure 2:**
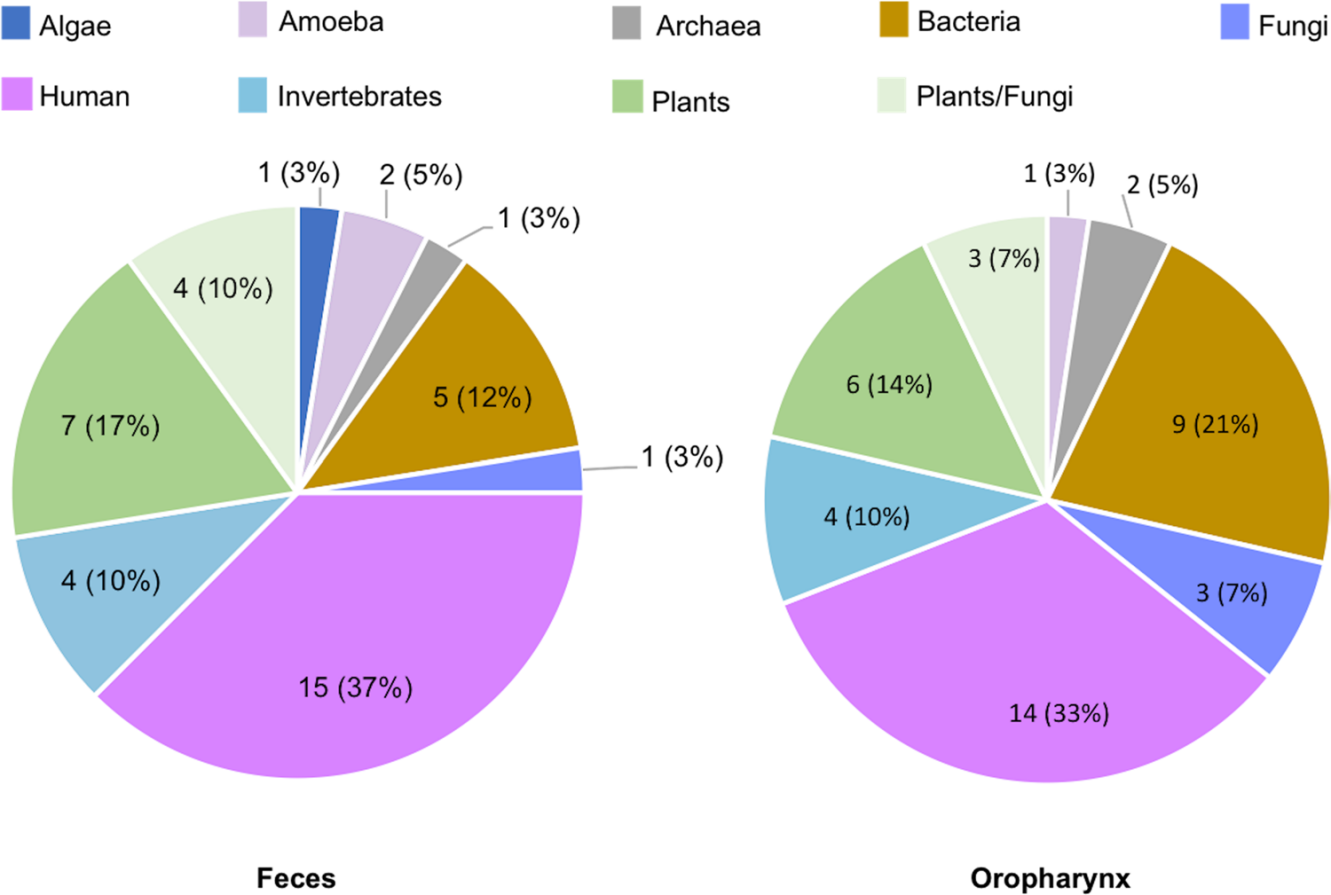
Hosts of the eukaryotic viral families identified in the infants’ fecal and oropharynx samples.

**Figure 3:**
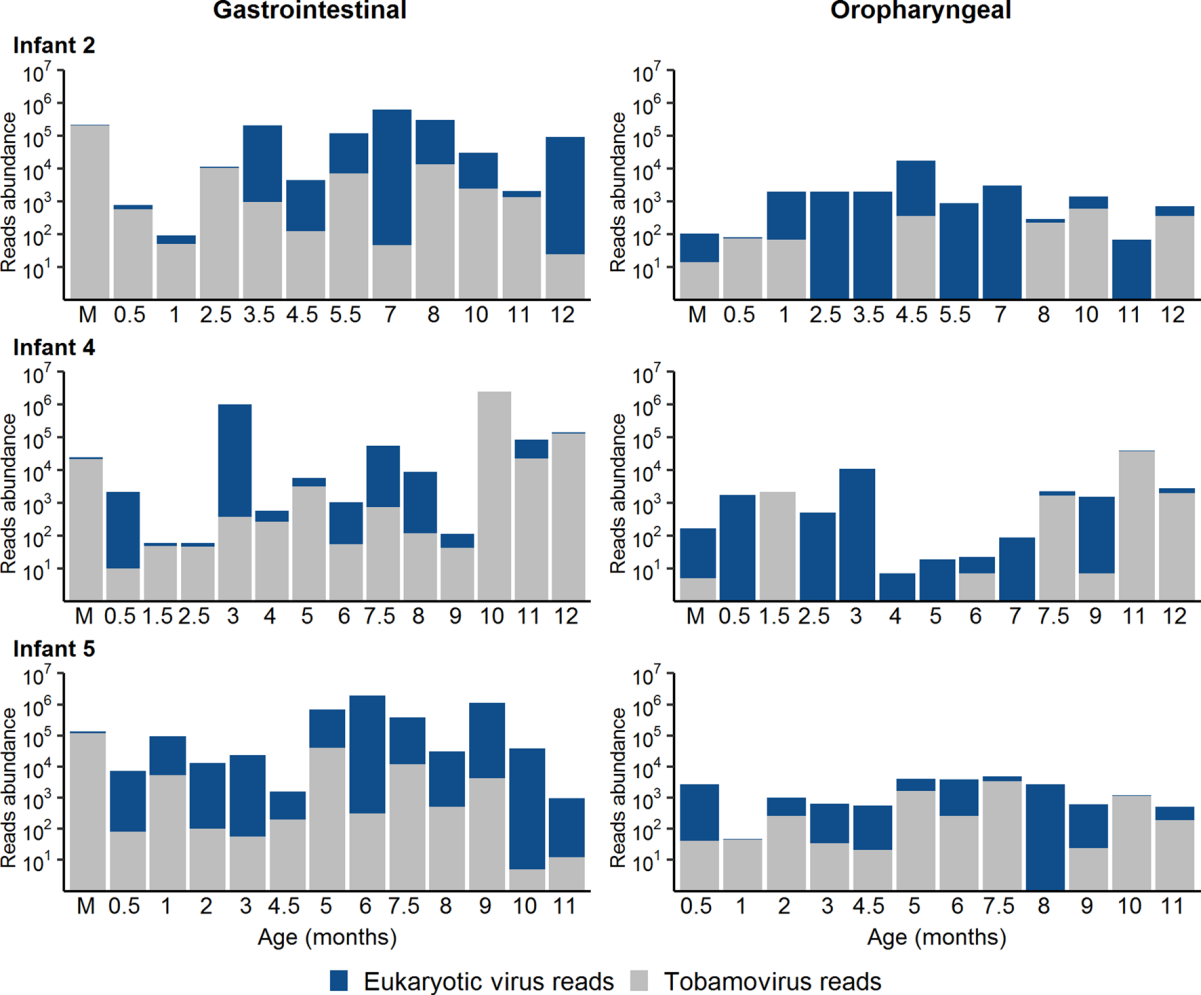
Abundance of tobamovirus and other eukaryotic virus of sequence reads assigned in the samples collected at the indicated age. Reads were normalized according to the total number of reads after quality filtering. M, sample from the corresponding mother. The oropharynx sample of the mother of infant 5 was not available.

A total of 37 oropharyngeal swabs and 38 stool samples were processed to look for both RNA and DNA viruses by next-generation sequencing using the NextSeq500 Illumina platform. An average of 4,781,260 sequence reads per gastrointestinal and 1,922,568 for oropharyngeal samples were generated. Complex eukaryotic viromes were detected in both types of samples, with viruses representing viral families that infect humans, insects, and plants, among other hosts (Fig. 2). It was surprising that plant viruses were the most common and abundant of all eukaryotic viruses detected, and were even present before the infants initiated a solid food regime (Fig. 3). This report focuses on the characterization of the diversity and dynamics of plant viruses in the study population. A complete description of the gut microbiome of these children will be published elsewhere (Taboada et al., in preparation).

### Plant viruses are common in the gastrointestinal tract during the first year of life

**Figure 4:**
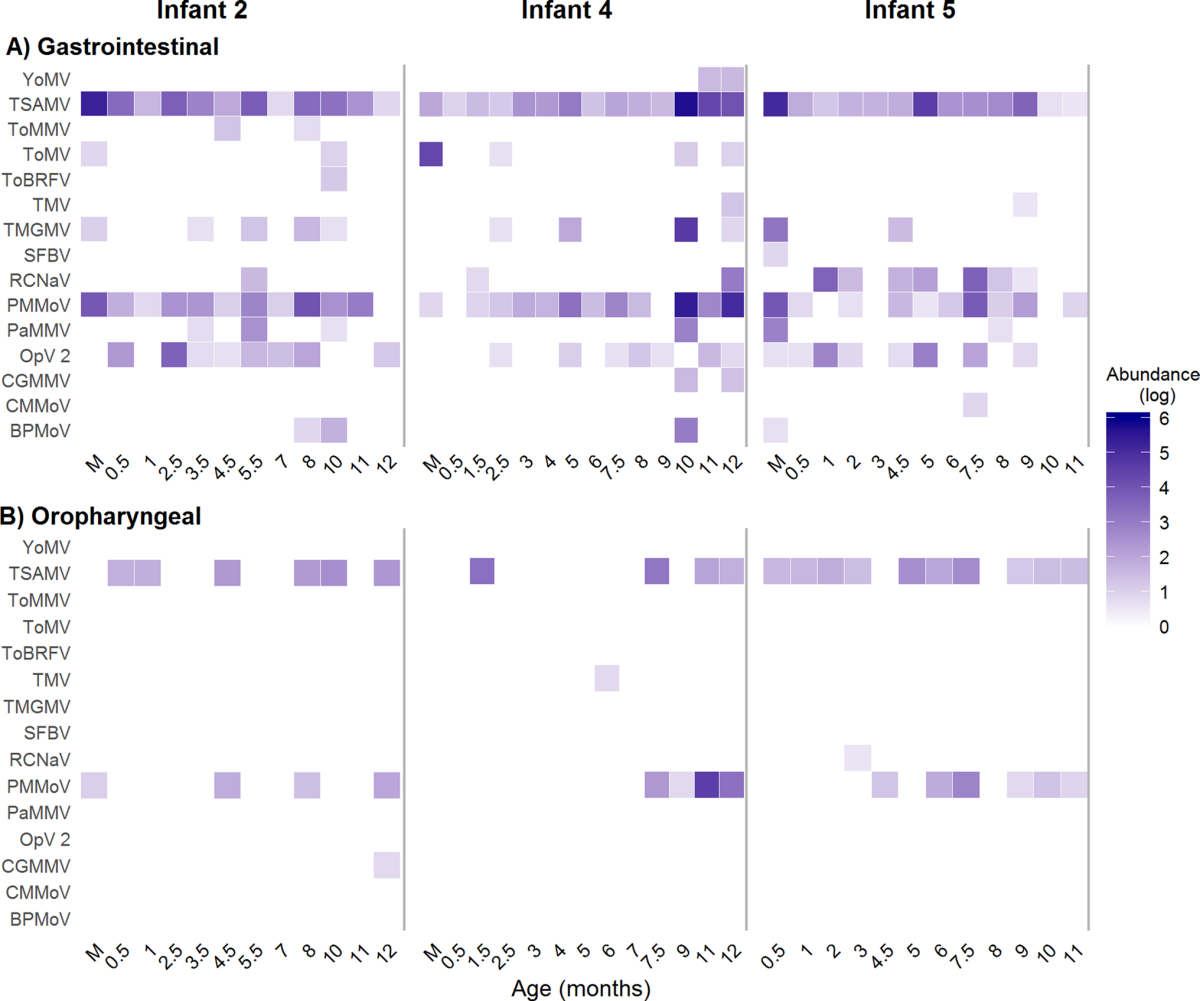
Abundance of tobamovirus species along the first year of life. (**A**) Gastrointestinal samples and (**B**) oropharyngeal samples. Sequence reads were normalized according to the total number of reads after quality filtering. The abundance is represented in a logarithmic scale (log10). M, sample from the corresponding mother. *YoMV* youcai mosaic virus, *TSAMV* tropical soda apple mosaic virus, *ToMMV* tomato mottle mosaic virus, *ToMV* tomato mosaic virus, *ToBRFV* tomato brown rugose fruit virus-israeli, *TMV* tobacco mosaic virus, *TMGMV* tobacco mild green mosaic virus, *SBFV* streptocarpus flower break virus, *RCNaV* rattail cactus necrosis-associated virus, *PMMoV* pepper mild mottle virus, *PaMMV* paprika mild mottle virus, *OpV2* opuntia tobamovirus 2, *CGMMoV* cucumber green mottle mosaic virus, *CMMoV* cactus mild mottle virus, *BPMV* bell pepper mottle virus.

The analysis of the gastrointestinal samples showed that at least one plant virus was detected in each of them. These viruses represented 13 different families (Table 3). Notably, the great majority of viral species belonged to the family *Virgaviridae*, specifically to the genus *Tobamovirus*, which was present in all samples. The sequence reads assigned to tobamoviruses in the samples collected from children accounted, in average, for 24.7% of the total eukaryotic viral reads, and in some samples represented up to 90% of these reads (Fig. 3). Fifteen different species of tobamoviruses were found; tropical soda apple mosaic virus (TSAMV), pepper mild mottle virus (PMMoV), and opuntia tobamovirus 2 (OpV2) were the most commonly identified species, being detected in 100% (38/38), 84% (32/38), and 61% (23/38) of the samples, respectively (Fig. 4A). Tobacco mild green mosaic virus (TMGMV) and rattail cactus necrosis-associated virus (RCNaV) were also commonly found. Of interest, tobamoviruses were found throughout the first year of life, even in samples collected 15 days after birth, and also in subsequent samples collected at times before the children started to ingest food or liquids different from breastmilk (Fig. 1). Regarding the fecal samples of the mothers, large amounts of tobamovirus sequences were also found, representing in average 90.6% of the total eukaryotic viral sequence reads present in the samples (Fig. 3).

**Table 3:** Abundance of sequence reads from the plant virus families identified.

	Feces	Oropharynx
Virus family	# reads (% of plant viruses total reads)	# reads (% of plant viruses total reads)
*Virgaviridae*	3,090,842 (99.83)	52,849 (94)
*Geminiviridae*	3,471 (0.112)	0
*Secoviridae*	511 (0.016)	0
*Alphaflexiviridae*	320 (0.01)	6 (0.010)
*Potyviridae*	723 (0.023)	342 (0.60)
*Tombusviridae*	173 (0.006)	4 (0.007)
*Bromoviridae*	34 (0.001)	4 (0.007)
*Betaflexiviridae*	33 (0.001)	0
*Luteoviridae*	16 (0.0005)	56 (0.10)
*Partitiviridae*	16 (0.0005)	671 (1.2)
*Endornaviridae*	20 (0.0006)	1,704 (3)
*Procedovirinae*	14 (0.0005)	0
*Caulimoviridae*	*0*	647 (1.1)

### Plant viruses are also common in the oropharynx of newborns and infants

Similar to what was found in the gastrointestinal samples, plant viruses were commonly identified in the oropharynx of children and their mothers, with 65% (24/37) of the samples being positive for these viruses. Nine different families of plant virus were detected in the oropharynx, with tobamoviruses being again the most prevalent (Table 3). Five species of tobamoviruses were identified, with PMMoV and TSAMV, being the most frequent, as observed in the fecal samples (Fig. 4B). These viruses were found in samples collected throughout the study, including the first trimester of life. The number of sequence reads assigned to tobamoviruses accounted, in average, for 46.40% of the total eukaryotic virus sequences found in children, and 6.27% in the samples collected from the mothers (Fig. 3).

### Tobamoviruses circulating in the population are highly diverse and dynamic

We further analyzed the sequence reads of the two most abundant and prevalent viruses (TSAMV and PMMoV) present in the gastrointestinal and oropharyngeal samples. We were able to assemble multiple contigs and 17 complete genomes, 9 of TSAMV, 6 of PMMoV, and one each of ToMV and RCNaV. In general, complete genomes were assembled from samples collected from children older than 5 months, although large contigs, of more than 500 bases, could be assembled from the gastrointestinal sample of infant 2 collected 15 days after birth (Table S1). In contrast, no complete genomes could be assembled from the oropharyngeal samples, although large contigs of more than 500 bases of TSAMV and PMMoV were assembled from various samples (Table S1).

**Figure 5:**
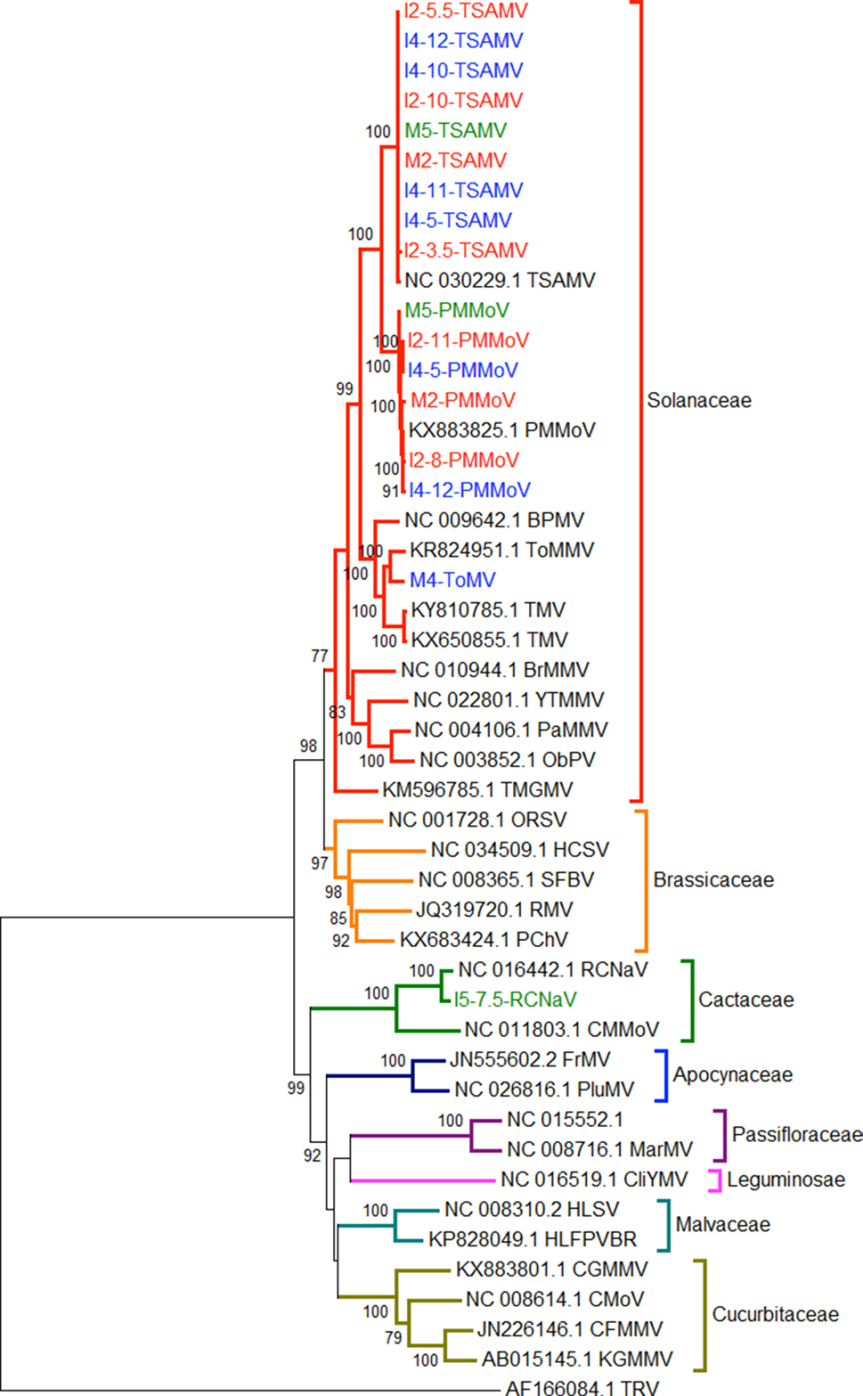
Phylogenetic tree of the complete *Tobamovirus* genomes (9 TSAMV, 6 PMMov, 1 ToMV and 1 RCNaV). On the right side, the plant family that serves as host for viruses in each clade is shown. The colors indicate the different children and mothers’ samples. Following the initial code, e.g., M2 for mother 2, and I2 for infant 2; the month at which the sample was collected is shown.

A phylogenetic analysis using the complete genome sequence of viruses in the genus *Tobamovirus* (Fig. 5) showed that they all segregated with sequences of viruses that infect the Solanaceae, a family of flowering plants that includes many edible plants, such as tomatoes, potatoes, eggplant, bell and chili peppers, among others, although also comprises vines, spices, shrubs, ornamentals, and weeds, such as the tropical soda apple. The only exception was RCNaV, whose full genomic sequence segregated with viruses in the family Cactaceae that are found in cactuses, like nopal.

**Figure 6:**
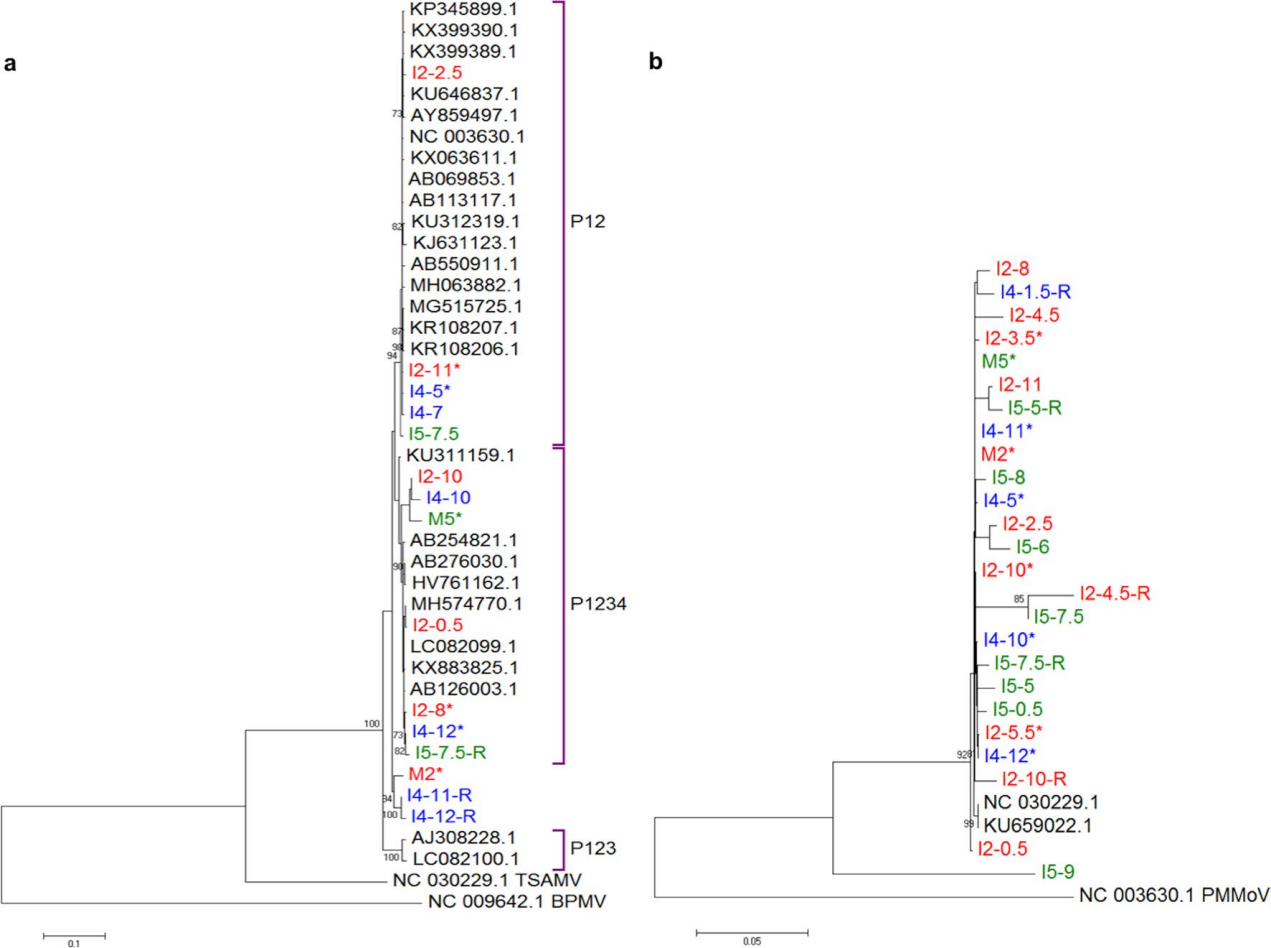
Phylogenetic trees of the complete genomes and partial sequences (contigs > 450 nt) of (**a**) PMMoV and (**b**) TSAMV. On the right side of the PMMoV tree, the pathotype of the reported reference strains is indicated. The colors indicate the different children and mothers’ samples. Following the initial code, e.g., M2 for mother 2, and I2 for infant 2; the month at which the sample was collected is shown. *, complete genomes. R, respiratory sample.

A more detailed phylogenetic analysis of PMMoV sequences, including 26 reference strains obtained from GenBank and the six complete genomes determined in this study, as well as six additional contigs between 467 and 5,846 bases long, showed that the reference strains grouped into three large clades that belong to pathotypes P12, P1234 or P123, which are classified based on the pathotypes reported for this virus: P1, P2, P3 and P4 that result from the ability of the viruses to overcome the plant resistance genes^13^. It can be observed that the contigs and complete genomes obtained in this study are distributed in two different pathotypes (Fig. 6a). It is of note that some sequences detected from different children fell in the same clade, e.g., I2_8 and I4_12 (evolution distance 0.003, see Table S2) or I2_11 and I4_5 (evolution distance 0.001); in contrast, in some instances, the nucleotide sequences of viruses detected in two consecutive samples of the same children were more distantly related, e.g., I2_10 and I2_11 (evolution distance 0.027), actually belonging to different pathotypes. In the case of TSAMV, only two complete genomes were annotated in GenBank prior to this work, and the nine genomes assembled in this study formed a new distinct clade, independent of the two previously reported sequences (Fig. 6b). In general, the phylogenetic analysis and evolution distances calculated for the different pairs of sequences (Tables S2 and S3) indicate that the frequent detection of PMMoV and TSAMV sequences in the gastrointestinal samples is derived from different variants of the viruses, suggesting that the children are continuously exposed to an extensive collection of high diversity tobamoviruses.

### RT-PCR detection of PMMoV and TSAMV

To confirm the presence of PMMoV and TSAMV genomic sequences in fecal and oropharyngeal samples isolated from infants, we analyzed the nucleic acids extracted from a subset of these samples by RT-PCR. In the fecal samples, 6 of 7 and 5 of 6 the samples tested were positive for PMMoV and TSAMV, respectively, while for throat samples none of 6 were positive for these viruses (data not shown). These data confirm the presence of these highly represented viruses in fecal samples; the failure of detection of the viruses in the throat samples might be due to the presence of more fragmented genomes (not a single complete genome could be assembled from these samples). No amplification was observed in fecal samples that were NGS-negative for these viruses (data not shown), indicating that the detection of PMMoV and TSMAV sequence reads by NGS was not of the result of contamination either from the library preparation or the sequencing procedure per se.

## Discussion

In this work, we report the frequent presence of plant virus sequence reads, particularly those corresponding to viruses belonging to the genus *Tobamovirus* in the *Virgaviridae* family, in the oropharynx and in the gastrointestinal tract of healthy children from a semi-rural community during their first year of life. Sequence reads of these viruses were detected as early as 2 weeks after birth in both sampled tracts and were found to be present in the majority of the samples analyzed, although there was clearly a larger abundance, diversity, and frequency of viruses in the gut as compared to oropharynx. Of interest, most of the PMMoV and TSAMV sequences characterized seem to correspond to variants of these viruses, suggesting a constant exposure of children to a highly diverse population of tobamoviruses. Interestingly, this is the first work that reports the presence of TSMAV in humans. This virus was described about a decade ago^14^, and its complete genome sequence was reported only recently^15^, what probably explains the previous lack of description of this virus in human feces. This is relevant considering that the diversity of plant viruses has been poorly studied^1,2^, suggesting the possibility that additional, and so far undetected plant viruses may be commonly circulating in the human gut.

We found no correlation in the abundance or in the virus species identified when the gastrointestinal and oropharyngeal samples were compared, indicating that the virus strains detected in the upper respiratory tract are not directly related to those found in the gut. The limited number of children studied does not allow to conclude whether the gender or the child delivery method influences the frequency and diversity of plant viruses detected, however, a follow up study with a larger number of children will address these points.

Few previous studies have characterized the gastrointestinal eukaryotic virome of children under 1 year of age^9–11,16^, and even fewer have been carried out in healthy children in the community^6–8^. In addition, our study represents the first longitudinal study with samples collected monthly. Only one previous study^6^ collected stool samples at six time points from birth to 2 years of age; in that study, carried out in a community of St. Louis, Missouri, USA, the fecal microbiome of 8 healthy infants (4 twin pairs) was characterized; viruses from the *Virgaviridae* as well as the *Alphaflexiviridae* and *Tombusviridae* families were found in a few samples obtained from children older than 18 months of age. The presence of geminivirus sequences was reported in a single sample collected from a child few days after birth^6^. In a transversal study, a single stool sample was collected from 10 healthy children between 4 and 10 days of age recruited from a community in Los Angeles, California^7^. In that study, several human viruses as well as arthropod and algae viruses were detected, but no plant viruses were reported. In an additional case–control study of malnourished infants/children carried out in Malawi in 20 twin pairs aged 0–30 months, plant viruses of the families *Geminiviridae* and *Begomoviridae* represented 3% of all assembled contigs^9^.

Some studies have detected the presence of *Virgaviridae* sequences in fecal samples of children with particular pathologies. Thus, in a study carried out in the Hunan province of China, 238 fecal samples were collected from pediatric patients (0–12.4 years of age) who were clinically diagnosed with mild or severe hand-foot-and-mouth disease^10^. In pools of samples from severe cases, 94% of the total viral reads were classified in the family *Virgaviridae*, however, these sequences were found in only three children, 5, 11 and 12-months old. In another study, a single fecal sample was collected from 35 South Asian children with acute flaccid paralysis; 3 children 1–13 years old were shown to have a *Tobamovirus*^9^. In an additional study carried out in Osaka, Japan, 5 fecal specimens from children 3–7 years old with diarrheal disease were characterized^17^; 4 species of *Tobamovirus* were detected in two samples, and in one sample, PMMoV reads outnumbered those corresponding to norovirus.

Studies characterizing the respiratory eukaryotic virome of children are even more scarce. Thus, in children with community acquired pneumonia (average age 2.36 ± 1.87 years) in Beijing Children’s Hospital, reads of more than 20 plant virus species were detected in 50% (25/50) of the nasopharyngeal aspirates^10^. Also, in a study carried out in Mexico, samples of children with respiratory tract infections resulted positive for plant virus sequences (8% in lower and 35% in upper respiratory tract)^18^. Whereas in a study in Japan no plant viruses were found in 3 nasopharyngeal aspirates of children 3–7 years old with respiratory disease^17^. As far as we know, there are no reports characterizing the eukaryotic virome of the respiratory tract or the oropharynx in children under 1 year of age using a next generation sequencing approach. In addition, our findings are in contrast with most previous studies, since plant virus sequences have been found with low frequency in both respiratory and gastrointestinal samples of children, and mostly in children older than 6 months of age, while in our study these viruses were found in the first weeks of age and remained throughout the first year of life.

Plant viruses have been commonly found in adult human feces, presumably from dietary origin^4,19^. In particular, PMMov has been detected in different geographic regions of the world, being found as frequently as in two-thirds of healthy human population^4^; in some cases, the fecal concentration of PMMoV was very high (10^9^ virions per gram of dry weight fecal matter) and it was suggested this might be the result of viral replication in the human gut^3,4,20^. In our case, the detection of tobamoviruses at early age stages, even before the children started eating food different from breastmilk, including infant formula or other liquids, suggests a different source origin for these viruses. None of the members of the families that participated in the study are smokers or work with tobacco products, suggesting that this is not likely to be the source of origin. On the other hand, traditional Mexican cuisine includes vegetables such as green peppers and tomatoes that are also known hosts of tobamoviruses. Some potential sources include the breast-feeding process, including transmission of the viruses from mother to child through breast milk; the handling of the mother and those in contact with the infant without properly hygiene measures; or airborne acquisition of the viruses. In children who have started infant formula or infusions early in life, before the introduction of solid food, a plausible origin for the viruses is the water used to prepare them. We are currently conducting a study to explore the main sources of plant viruses in the children gut, and the transmission pathways.

In this study the prevalence of PMMoV and TSAMV in both oropharynx and gastrointestinal samples is extraordinarily high compared to other plant viruses and even with other *Tobamovirus* species; RCaNV, OpV2, ToMV, and TMGMV were also frequently detected in feces, although to a lesser extent. It has been reported that tobamoviruses are highly stable under diverse environmental conditions, in fact PMMoV has been suggested as a quality water indicator, since its abundance correlates with the amount of fecal contamination of water^20^. This opens up the possibility of fecal contamination of water or food as another source for these viruses in the infants. The high frequency of detection of tobamoviruses could also be related to the abundance and diversity of edible plants in the Solanaceae family that are hosts of these viruses and that are commonly consumed in the studied community. These two factors could influence the continuous appearance of tobamoviruses in the human gastrointestinal tract and oropharynx. The reason for the high detection rate of tobamovirus sequences in our study as compared to previous studies in children is unclear. Whether it is related to methods used to prepare or analyze the samples, the depth of sequencing, the environmental conditions of the setting, or the socio-cultural or demographic characteristics of the population will require further studies.

The fact that fragments of viral genomes are detected by NGS does not mean that they are associated to infectious virus; however, the fact that 15 different complete genomes of PMMoV and TSAMV could be assembled strongly suggests the presence of infectious virus particles at least in some samples. Of interest, it has been shown that PMMoV present in adult feces was indeed infectious when inoculated into host plants^4,19^, and it has been debated whether plant viruses can replicate in the human host, particularly in the gut^3^. In this regard, it is surprising that in some fecal samples of pre-weaning infants the number of reads was high enough to assemble complete genomes and, in some cases, this number was comparable to that found in the mothers’ fecal samples (Fig. 2). Besides, some of these viruses were continuously present in the infants' gut throughout their first year of life. Although this does not prove that the viruses are replicating, it guarantees the investigation of this possibility using in vitro models, such as enteroids^21^ or animal models.

It is now clear that the bacterial communities in the gastrointestinal tract play a critical role on different human developmental pathways early in life^22^. In this regard, it is of great interest the considerable exposure that infants can have to plant viruses during the first year of life, as shown by the high frequency of detection of tobamoviruses in both the oropharynx (65%) and fecal (100%) samples starting at least 2 weeks after birth. This raises the question of whether these viruses could have an impact in the maturation of the infant’s immune system or in their healthy development, directly or through interactions with other components of the microbiota, as has been observed for norovirus in a mouse model^23^. This possibility needs to be investigated.

## Methods

### Sampling

The study was conducted in the semi-rural community of Xoxocotla, Morelos, about 70 miles south of Mexico City, in collaboration with the Ministry of Health of the State of Morelos. The women who attended the pregnancy control services at the Xoxocotla community Health Center were voluntarily enrolled in the study in their third trimester of pregnancy, and fecal and oropharynx samples were collected from them about 1 month before delivery. The criteria for inclusion were healthy pregnant women in their third trimester of a normal pregnancy. In this study, we selected the three mother/infant pairs that had the most complete sampling record throughout the year of study. The oropharynx sample of the mother of infant 5 was not available. Oropharynx swabs and fecal samples were collected monthly between March 2015 and June 2016 from three children (Fig. 1), with no apparent clinical signs of disease. The inclusion criteria for infants were children born at term (38–42 weeks of pregnancy), clinically healthy, and with no congenital diseases. Sterile plastic containers with screw caps were used to collect stool samples from diapers. The diapers were placed inverted in the babies to avoid absorption of the samples. Dry sterile swabs (rayon-tipped, BD BBL Culture swab) were used to collect samples from the back of the throat and were placed in vials containing 1 ml of viral transport medium (Microtest M4-RT, Remel). The samples were maintained at 4 °C and transported to the laboratory on the same day of collection. Both fecal and oropharynx samples were kept at − 20 °C for 1 week and then stored at − 70 °C, until use. During the study, the mothers reported that antibiotics were not used, as well as the absence of respiratory symptoms or gastrointestinal disease in children during the study period.

### Nucleic acid isolation and sequencing

Nucleic acids were extracted basically as previously described^24^. For stool samples, a 10% homogenate was prepared using a bead beater (Biospec Products, USA). The mixture contained 100 mg of 150–212 μm glass beads (Sigma, USA) in a final volume of 10% chloroform in 1 ml of phosphate-buffered saline (PBS). After homogenization, the samples were centrifuged at 2,000×*g* to remove large debris, and the supernatants were filtered in Spin-X 0.45 μm pore filters (Costar, NY) at 10,000×*g* for 10 to 20 min. Filtered samples (400 μl) were treated with Turbo DNase (4U) (Ambion, USA) and RNAse (2U) (Sigma, USA) for 30 min at 37 °C, and nucleic acids were extracted using the PureLink Viral RNA/DNA extraction kit according to the manufacturer’s instructions (Invitrogen, USA). The extracted nucleic acids were eluted in nuclease-free water, aliquoted, and stored at − 70 °C until further use. For respiratory samples, the preparation consisted in removal of cellular debris by centrifugation at 2,000×*g* for 5 min. The supernatant was then processed by filtration through Spin-X 0.45 μm pore filters (Costar, NY), followed by the protocol described above.

The extracted nucleic acids were amplified basically as previously described^24,25^. First, the RNA was reverse transcribed using SuperScript III reverse transcriptase (Invitrogen, USA) using primer A (5′-GTTTCCCAGTAGGTCTCN9-3′). The second strand DNA was generated by two consecutive rounds of synthesis using Sequenase 2.0 (USB, USA). The obtained DNA was amplified using Phusion High fidelity polymerase (Finnzymes) using primer B (5′-GTTTCCCAGTAGGTCTC-3′) by 10 cycles of the program: 30 s at 94 °C, 1 min at 50 °C and 1 min at 72 °C. The amplified DNA was cleaned by ZYMO DNA Clean and Concentration-5 kit. Sequencing libraries were prepared using a Nextera XT DNA library preparation kit (Illumina). Samples were tagged, multiplexed and sequencing was performed by pair-end 72 cycles of nucleotide extension in a NextSeq500 Illumina platform.

### RT-PCR

RNA was isolated from fecal and respiratory samples as described above. Reverse transcription was performed with M-MLV Reverse Transcriptase Kit (Thermo Fisher Scientific, USA) using the reverse specific primers for each virus species. For PCR amplification Taq DNA Polymerase (ThermoFisher, USA) was used, using both forward and reverse primers for each virus species. The primers were designed to match highly conserved genomic regions of the corresponding virus species. For PMMoV, the forward and reverse primers used were 5′-GGAAAACGCCTACACAGATCG-3′ (nucleotides 4,151 to 4,171, corresponding to PMMoV, accession number MG515725.1) and 5′-GTACGCACAATTGTTCAACG-3′ (nucleotides 4,782–4,763), respectively. For TSAMV, the oligonucleotides forward 5′-CAGACACATGATTAAGCAGC-3′ (nucleotides 3,966–3,985 of TSAMV, corresponding to accession number KU659022.1) and reverse 5′-CCTTCCACAAAAGTACCCG-3′ (nucleotides 4,626–4,608) were used.

### Sequence analysis and assembly of viral genomes

Reads of each sample were preprocessed as previously reported including quality control, duplication removal and human and ribosomal filtering^26^. The preprocessed reads of each sample were mapped against a database of all complete genomes of the *Virgaviridae* family reported in Genbank at June 2018, using BBMap v38.26 at 70% identity. Hit reads at the species level were obtained to perform de novo assemblies using IDBA v.1.1.3, selecting only contigs longer than 400 bases. Consensus sequences were generated in those cases in which complete genomes were not obtained. To this end, the sequence reads were mapped only to a single reference genome. Next, Samtools v.1.9 was used to obtain a first consensus sequence, used as reference to map again the reads using Bowtie2 v2.3.4.3 to generate a final consensus sequence. Afterwards, all contigs were corrected by a variant calling process to eliminate assembly or consensus errors. Finally, each contig or complete genome assembled was taxonomical verified by a Blastn.

### Phylogenetic analysis

All complete genome sequences of viruses in the *Tobamovirus* genus reported in GenBank in June 2018 were included, as well as sequences of individual species of interest. A subset of representative sequences was obtained by CD-HIT analysis at a 95% identity, which was used to make a multiple base alignment using ClustalW. The evolution model that best fit each alignment was determined using MEGA v5. The complete and partial sequences were added to the base alignment using Mafft v7. Finally, phylogenetic trees were constructed using the Maximum Likelihood method with the evolution model obtained of base alignment with 1,000 bootstrap.

### Statistical analysis

The evolutionary distances of the assembled sequences were calculated with the MEGA v6 software using the evolution model that best fit each alignment.

### Ethical considerations

The protocol used in this study was conducted under the ethical principles and approval of both the Mexican Ethics and Research Commission of the Health Ministry of the State of Morelos and the Ethics in Research Commission of the Faculty of Medicine (Project # 088/2014) of the National University of Mexico (UNAM). It was also approved by the Bioethics Committee of the Institute of Biotechnology (Project # 261) of UNAM. The guidelines of the Committees are based on the Mexican Official Norm (Norma Oficial Mexicana NOM-012-SSA3-2007), which regulates the ethical principles of every research on humans, as well as on the Declaration of Helsinki, regarding research studies involving humans, approved by the World Health Organization. Written informed consent was obtained from each parent or guardian prior to enrollment.

## Supplementary information

Supplementary Tables.

## Data Availability

The database of the Bioproject PRJNA592261, which contains all the biosamples and complete genomes, is available at NCBI.
