# Supplementary material for: Tobamoviruses can be frequently present in the oropharynx and gut of infants during their first year of life
Source: Sci Rep. 2020 Aug 12;10:13595. doi: 10.1038/s41598-020-70684-w (PMC7423923; doi:10.1038/s41598-020-70684-w)
Supplement: Supplementary file 1 — Supplementary Tables. [file 41598_2020_70684_MOESM1_ESM.docx]

**Supplementary Information**

**Tobamoviruses can be frequently present in the oropharynx and gut of infants during their first year of life**

Yarenci Aguado-García^1†^, Blanca Taboada^1†^, Xaira Rivera-Gutiérrez^1^, Pavel Iša^1^, Angélica Serrano^2^, Patricia Morán^2^, Liliana Rojas^2^, Horacio Pérez^2^, Susana López^1^, Javier Torres^3*^, Cecilia Ximenez^2*^, Carlos F. Arias^1*^.

^1^Instituto de Biotecnología, Universidad Nacional Autónoma de México, Av. Universidad 2001, Cuernavaca, Morelos 62210; ^2^Unidad de Investigación en Medicina Experimental, Facultad de Medicina, Universidad Nacional Autónoma de México, Cuauhtémoc 06726 Ciudad de México; ^3^Unidad de Investigación Médica en Enfermedades Infecciosas y Parasitarias, Hospital Pediatría, Centro Médico Nacional Siglo XXI, Instituto Mexicano del Seguro Social, Cuauhtémoc 06726, Ciudad de México, Mexico.

Yarenci Aguado-García: yarenci@ibt.unam.mx

Blanca Taboada: btaboada@ibt.unam.mx

Xaira Rivera-Gutiérrez: xaira.rivera@mail.ibt.unam.mx

Pavel Isa: pavel@ibt.unam.mx

Angélica Serrano: anseva_31@yahoo.com.mx

Patricia Morán: patricia_morans@yahoo.com.mx

Liliana Rojas: lhily@yahoo.com

Horacio Pérez: daztlan13@gmail.com

Susana López: susana@ibt.unam.mx

Javier Torres: uimeip@gmail.com

Cecilia Ximénez: cximenez2005@yahoo.com.mx

Carlos F. Arias: arias@ibt.unam.mx

**Table S1.** Statistics of the contigs longer than 450 nucleotides included in the phylogenetic trees.

| **Virus** | **Tract** | **Child** | **Contig ID** | **Age (months)** | **Size (b)** | **Mapped reads** | **Deep coverage** |
| --- | --- | --- | --- | --- | --- | --- | --- |
| **PMMoV** | G | **2** | M2* | M | 6,208 | 7,182 | 87.81 |
|  | G |  | I2-0.5 | 0.5 | 584 | 40 | 4.67 |
|  | G |  | I2-2.5 | 2.5 | 467 | 268 | 39.07 |
|  | G |  | I2-8* | 8 | 6,244 | 10,744 | 103.03 |
|  | G |  | I2-10 | 10 | 808 | 56 | 4.57 |
|  | G |  | I2-11* | 11 | 6,036 | 1,075 | 13.32 |
|  |  |  |  |  |  |  |  |
|  | G | **4** | I4-5* | 5 | 6,254 | 1,917 | 22.87 |
|  | G |  | I4-7 | 7 | 5,785 | 639 | 7.73 |
|  | G |  | I4-10 | 10 | 5,846 | 268,637 | 3,377.81 |
|  | G |  | I4-12* | 12 | 6,259 | 115,884 | 1,389.43 |
|  | R |  | I4-11-R | 11 | 6,151 | 40,877 | 465.19 |
|  | R |  | I4-12-R | 12 | 2,777 | 13,120 | 330.72 |
|  |  |  |  |  |  |  |  |
|  | G | **5** | M5* | M | 6,160 | 8,754 | 126.13 |
|  | G |  | I5-7.5 | 7.5 | 5,268 | 6,156 | 84.71 |
|  | R |  | I5-7.5-R | 7.5 | 1,458 | 328 | 16.37 |
|  |  |  |  |  |  |  |  |
| **TSAMV** | G | **2** | M2* | M | 6,293 | 196,083 | 2,366.77 |
|  | G |  | I2-0.5 | 0.5 | 1,298 | 252 | 13.88 |
|  | G |  | I2-2.5 | 2.5 | 2,858 | 5,607 | 137.33 |
|  | G |  | I2-3.5* | 3.5 | 6,240 | 708 | 8.45 |
|  | G |  | I2-4.5 | 4.5 | 680 | 48 | 4.50 |
|  | G |  | I2-5.5* | 5.5 | 6,283 | 6,204 | 76.04 |
|  | G |  | I2-8 | 8 | 4,546 | 2,704 | 41.64 |
|  | G |  | I2-10* | 10 | 6,315 | 1,990 | 23.28 |
|  | G |  | I2-11 | 11 | 983 | 60 | 4.14 |
|  | R |  | I2-4.5-R | 4.5 | 484 | 175 | 26.61 |
|  | R |  | I2-10-R | 10 | 2,409 | 497 | 14.44 |
|  |  |  |  |  |  |  |  |
|  | G | **4** | I4-5* | 5 | 6,065 | 1,223 | 16.61 |
|  | G |  | I4-10* | 10 | 6,296 | 2,153,878 | 20,830.65 |
|  | G |  | I4-11* | 11 | 6,305 | 21,626 | 201.45 |
|  | G |  | I4-12* | 12 | 6,256 | 11,439 | 154.87 |
|  | R |  | I4-1.5-R | 1.5 | 1,377 | 2,203 | 119.73 |
|  |  |  |  |  |  |  |  |
|  | G | **5** | M5* | M | 6,320 | 127,311 | 1,536.82 |
|  | G |  | I5-0.5 | 0.5 | 506 | 47 | 5.49 |
|  | G |  | I5-5 | 5 | 4,927 | 39,833 | 565.92 |
|  | G |  | I5-6 | 6.5 | 892 | 293 | 24.61 |
|  | G |  | I5-7.5 | 7.5 | 1,474 | 334 | 13.66 |
|  | G |  | I5-8 | 8.5 | 3,623 | 413 | 7.98 |
|  | G |  | I5-9 | 9 | 4,800 | 4,032 | 58.8 |
|  | R |  | I5-5-R | 5 | 1,769 | 3,334 | 131.93 |
|  | R |  | I5-7.5-R | 7.5 | 4,584 | 3,603 | 55.06 |
|  |  |  |  |  |  |  |  |

* G= Gastrointestinal; R=Respiratory; PMMoV=Pepper mild mottle virus; TSAMV= Tropical soda apple mosaic virus.

**Table S2.** Pair-wise evolutive distances for the Pepper mild mottle virus (PMMoV) sequences.

|  | **1** | **2** | **3** | **4** | **5** | **6** | **7** | **8** | **9** | **10** | **11** | **12** | **13** | **14** | **15** |
| --- | --- | --- | --- | --- | --- | --- | --- | --- | --- | --- | --- | --- | --- | --- | --- |
| **1. M2_6208*** | 0 |  |  |  |  |  |  |  |  |  |  |  |  |  |  |
| **2. I2_0.5_584** | 0.018 | 0 |  |  |  |  |  |  |  |  |  |  |  |  |  |
| **3. I2_2.5_467** | 0.044 | NA | 0 |  |  |  |  |  |  |  |  |  |  |  |  |
| **4. I2_8_6244*** | 0.029 | 0.003 | 0.040 | 0 |  |  |  |  |  |  |  |  |  |  |  |
| **5. I2_10_808** | 0.033 | NA | NA | 0.006 | 0 |  |  |  |  |  |  |  |  |  |  |
| **6. I2_11_6036*** | 0.031 | 0.023 | 0.007 | 0.029 | 0.027 | 0 |  |  |  |  |  |  |  |  |  |
| **7. I4_5_6254*** | 0.030 | 0.025 | 0.007 | 0.028 | 0.025 | 0.001 | 0 |  |  |  |  |  |  |  |  |
| **8. I4_7_5485** | 0.031 | 0.025 | 0.007 | 0.028 | 0.027 | 0.002 | 0.002 | 0 |  |  |  |  |  |  |  |
| **9. I4_10_5846** | 0.048 | 0.016 | 0.059 | 0.028 | 0.018 | 0.041 | 0.043 | 0.040 | 0 |  |  |  |  |  |  |
| **10. I4_12_6259*** | 0.030 | 0.003 | 0.042 | 0.003 | 0.004 | 0.029 | 0.028 | 0.028 | 0.027 | 0 |  |  |  |  |  |
| **11. I4_11_6151_R** | 0.024 | 0.018 | 0.035 | 0.027 | 0.037 | 0.029 | 0.029 | 0.030 | 0.048 | 0.028 | 0 |  |  |  |  |
| **12. I4_12_2777_R** | 0.029 | NA | 0.035 | 0.037 | 0.036 | 0.041 | 0.040 | 0.041 | 0.052 | 0.038 | 0.007 | 0 |  |  |  |
| **13. M5_6160*** | 0.045 | 0.012 | 0.032 | 0.035 | 0.006 | 0.036 | 0.038 | 0.035 | 0.030 | 0.036 | 0.046 | 0.049 | 0 |  |  |
| **14. I5_7.5_5268** | 0.032 | 0.029 | 0.009 | 0.029 | 0.031 | 0.006 | 0.007 | 0.006 | 0.035 | 0.030 | 0.026 | 0.035 | 0.028 | 0 |  |
| **15. I5_7.5_1458_R** | 0.035 | NA | 0.040 | 0.008 | NA | 0.036 | 0.033 | 0.036 | 0.025 | 0.005 | 0.037 | 0.042 | 0.027 | 0.037 | 0 |

* complete genome sequences.

Pair-wise distances were calculated by using Tamura-Nei Model, with MEGA6 software.

**Table S3.** Pair-wise evolutive distances for the Tropical soda apple mosaic virus (TSAMV) sequences.

|  | **1** | **2** | **3** | **4** | **5** | **6** | **7** | **8** | **9** | **10** | **11** | **12** | **13** | **14** | **15** | **16** | **17** | **18** | **19** | **20** | **21** | **22** | **23** | **24** | **25** |
| --- | --- | --- | --- | --- | --- | --- | --- | --- | --- | --- | --- | --- | --- | --- | --- | --- | --- | --- | --- | --- | --- | --- | --- | --- | --- |
| **1. M2_6300*** | 0 |  |  |  |  |  |  |  |  |  |  |  |  |  |  |  |  |  |  |  |  |  |  |  |  |
| **2. I2_0.5_1298** | 0.002 | 0 |  |  |  |  |  |  |  |  |  |  |  |  |  |  |  |  |  |  |  |  |  |  |  |
| **3. I2_2.5_2858** | 0.009 | 0.019 | 0 |  |  |  |  |  |  |  |  |  |  |  |  |  |  |  |  |  |  |  |  |  |  |
| **4. I2_3.5_6240*** | 0.002 | 0.003 | 0.011 | 0 |  |  |  |  |  |  |  |  |  |  |  |  |  |  |  |  |  |  |  |  |  |
| **5. I2_4.5_680** | 0.013 | 0.017 | 0.023 | 0.015 | 0 |  |  |  |  |  |  |  |  |  |  |  |  |  |  |  |  |  |  |  |  |
| **6. I2_5.5_6283*** | 0.002 | 0.004 | 0.011 | 0.004 | 0.016 | 0 |  |  |  |  |  |  |  |  |  |  |  |  |  |  |  |  |  |  |  |
| **7. I2_8_4545** | 0.008 | 0.005 | 0.013 | 0.008 | 0.018 | 0.008 | 0 |  |  |  |  |  |  |  |  |  |  |  |  |  |  |  |  |  |  |
| **8. I2_10_6315*** | 0.002 | 0.003 | 0.010 | 0.002 | 0.016 | 0.002 | 0.007 | 0 |  |  |  |  |  |  |  |  |  |  |  |  |  |  |  |  |  |
| **9. I2_11_983** | 0.004 | NA | NA | 0.006 | NA | 0.006 | 0.002 | 0.004 | 0 |  |  |  |  |  |  |  |  |  |  |  |  |  |  |  |  |
| **10. I2_4.5_484** | 0.021 | NA | 0.061 | 0.021 | NA | 0.024 | NA | 0.021 | NA | 0 |  |  |  |  |  |  |  |  |  |  |  |  |  |  |  |
| **11. I2_10_2409_R** | 0.009 | 0.018 | 0.029 | 0.010 | NA | 0.011 | 0.010 | 0.009 | 0.004 | 0.021 | 0 |  |  |  |  |  |  |  |  |  |  |  |  |  |  |
| **12. I4_5_6065*** | 0.001 | 0.003 | 0.009 | 0.003 | 0.015 | 0.003 | 0.006 | 0.001 | 0.004 | 0.021 | 0.010 | 0 |  |  |  |  |  |  |  |  |  |  |  |  |  |
| **13. I4_10_6297*** | 0.001 | 0.003 | 0.010 | 0.003 | 0.015 | 0.002 | 0.008 | 0.002 | 0.004 | 0.024 | 0.010 | 0.002 | 0 |  |  |  |  |  |  |  |  |  |  |  |  |
| **14. I4_11_6306*** | 0.000 | 0.002 | 0.009 | 0.002 | 0.013 | 0.002 | 0.007 | 0.005 | 0.004 | 0.021 | 0.009 | 0.001 | 0.001 | 0 |  |  |  |  |  |  |  |  |  |  |  |
| **15. I4_12_6256*** | 0.001 | 0.004 | 0.010 | 0.003 | 0.016 | 0.000 | 0.007 | 0.002 | 0.006 | 0.024 | 0.010 | 0.002 | 0.001 | 0.002 | 0 |  |  |  |  |  |  |  |  |  |  |
| **16. I4_1.5_1337_R** | 0.008 | 0.005 | 0.027 | 0.009 | 0.015 | 0.011 | 0.007 | 0.010 | NA | NA | 0.038 | 0.009 | 0.010 | 0.008 | 0.011 | 0 |  |  |  |  |  |  |  |  |  |
| **17. M5_6320*** | 0.001 | 0.002 | 0.010 | 0.002 | 0.013 | 0.002 | 0.008 | 0.004 | 0.004 | 0.021 | 0.009 | 0.001 | 0.002 | 0.000 | 0.002 | 0.008 | 0 |  |  |  |  |  |  |  |  |
| **18. I5_0.5_506** | 0.004 | NA | 0.012 | 0.006 | NA | 0.004 | 0.004 | 0.004 | 0.005 | NA | 0.008 | 0.006 | 0.004 | 0.004 | 0.004 | NA | 0.004 | 0 |  |  |  |  |  |  |  |
| **19. I5_5_4926** | 0.010 | 0.010 | 0.014 | 0.011 | 0.029 | 0.009 | 0.012 | 0.009 | 0.023 | 0.044 | 0.014 | 0.009 | 0.010 | 0.009 | 0.008 | 0.018 | 0.009 | 0.004 | 0 |  |  |  |  |  |  |
| **20. I5_6_892** | 0.014 | NA | 0.005 | 0.017 | NA | 0.015 | 0.018 | 0.014 | NA | NA | NA | 0.014 | 0.014 | 0.014 | 0.014 | NA | 0.015 | NA | 0.017 | 0 |  |  |  |  |  |
| **21. I5_8_3623** | 0.005 | 0.006 | 0.011 | 0.006 | 0.031 | 0.005 | 0.006 | 0.005 | 0.011 | 0.036 | 0.014 | 0.005 | 0.006 | 0.005 | 0.005 | 0.018 | 0.005 | 0.010 | 0.014 | 0.026 | 0 |  |  |  |  |
| **22. I5_9_4800** | 0.144 | 0.112 | 0.148 | 0.145 | 0.162 | 0.146 | 0.147 | 0.144 | 0.170 | 0.163 | 0.154 | 0.148 | 0.146 | 0.143 | 0.145 | 0.143 | 0.145 | 0.176 | 0.146 | 0.160 | 0.150 | 0 |  |  |  |
| **23. I5_7.5_1474** | 0.024 | 0.183 | 0.041 | 0.026 | 0.206 | 0.026 | 0.050 | 0.025 | NA | 0.019 | 0.010 | 0.024 | 0.024 | 0.024 | 0.025 | 0.105 | 0.024 | NA | 0.040 | 0.010 | 0.011 | 0.188 | 0 |  |  |
| **24. I5_5_1769_R** | 0.012 | 0.009 | 0.024 | 0.015 | NA | 0.012 | 0.020 | 0.012 | 0.002 | NA | 0.034 | 0.014 | 0.013 | 0.012 | 0.012 | NA | 0.012 | 0.045 | 0.024 | NA | 0.020 | 0.179 | NA | 0 |  |
| **25. I5_7.5_4584_R** | 0.006 | 0.018 | 0.007 | 0.008 | 0.018 | 0.006 | 0.008 | 0.012 | 0.004 | 0.021 | 0.009 | 0.007 | 0.005 | 0.008 | 0.005 | 0.023 | 0.007 | 0.004 | 0.012 | 0.016 | 0.010 | 0.165 | 0.027 | 0.019 | 0 |

* complete genome sequences.

Pair-wise distances were calculated by using Tamura-Nei model, with MEGA6 software.
